# Sugar-Sweetened Beverages and Cardiometabolic Health: An Update of the Evidence

**DOI:** 10.3390/nu11081840

**Published:** 2019-08-08

**Authors:** Vasanti S. Malik, Frank B. Hu

**Affiliations:** 1 Department of Nutritional Sciences, Faculty of Medicine, University of Toronto, 1 King’s College Circle, Toronto, ON M5S 1A8, Canada; 2 Department of Nutrition, Harvard T.H. Chan School of Public Health, 665 Huntington Avenue, Boston, MA 02115, USA; 3 Department of Epidemiology, Harvard T.H. School of Public Health, Boston, MA 02115, USA; 4 Channing Division of Network Medicine, Brigham and Women’s Hospital and Harvard Medical School, Boston, MA 02115, USA

**Keywords:** sugar-sweetened beverages, metabolic syndrome, weight gain, type 2 diabetes, cardiovascular disease, cardiometabolic risk

## Abstract

Sugar-sweetened beverages (SSBs) have little nutritional value and a robust body of evidence has linked the intake of SSBs to weight gain and risk of type 2 diabetes (T2D), cardiovascular disease (CVD), and some cancers. Metabolic Syndrome (MetSyn) is a clustering of risk factors that precedes the development of T2D and CVD; however, evidence linking SSBs to MetSyn is not clear. To make informed recommendations about SSBs, new evidence needs to be considered against existing literature. This review provides an update on the evidence linking SSBs and cardiometabolic outcomes including MetSyn. Findings from prospective cohort studies support a strong positive association between SSBs and weight gain and risk of T2D and coronary heart disease (CHD), independent of adiposity. Associations with MetSyn are less consistent, and there appears to be a sex difference with stroke with greater risk in women. Findings from short-term trials on metabolic risk factors provide mechanistic support for associations with T2D and CHD. Conclusive evidence from cohort studies and trials on risk factors support an etiologic role of SSB in relation to weight gain and risk of T2D and CHD. Continued efforts to reduce intake of SSB should be encouraged to improve the cardiometabolic health of individuals and populations.

## 1. Introduction

Metabolic syndrome (MetSyn) is known as a clustering of interrelated risk factors for type 2 diabetes (T2D) and cardiovascular disease (CVD) that occur together more often than by chance alone. Although there is some confusion regarding the clinical definition of MetSyn and whether it is a unique syndrome or a mixture of unrelated phenotypes, the most widespread consensus for a diagnosis is the presence of at least three of five risk factors including hyperglycemia, raised blood pressure, elevated triglyceride levels, low high-density lipoprotein cholesterol levels, and central adiposity [1]. Given the complexity of the definition, the prevalence of MetSyn is difficult to estimate; however, data on individual risk factors suggest that MetSyn is rising across the globe in parallel with obesity trends. It was estimated that 23% of adults in the United States (US) (~50 million) have MetSyn [2,3]. This figure was relatively constant over recent years despite population-level increases in hyperglycemia and waist circumference because of decreases in hypertriglyceridemia and elevated blood pressure corresponding to medication use [4]. However, the burden of MetSyn remains high in the US and is rising in low- and middle-income countries (LMICs) [3]. This is of great concern, since individuals with MetSyn are at twice the risk of developing CVD and have a five-fold higher risk of developing T2D over the next 5–10 years [1]. Preventing or reversing MetSyn could, therefore, be an effective way to stem the rising tide of T2D and CVD.

Sugar-sweetened beverages (SSBs) are the largest source of added sugar in the diet. They include carbonated and non-carbonated soft drinks, fruit drinks, and sports drinks that contain added caloric sweeteners, and they are low in nutritional quality. To date, a large body of evidence supports a strong link between intake of SSBs and weight gain [5] and risk of T2D [6], which is the basis of many dietary guidelines and policies targeting SSBs [7]. Emerging evidence suggests that SSBs are also an important risk factor for cardiovascular diseases and related risk factors [8,9,10,11,12]. However, evidence linking SSBs to MetSyn is not clear. For clinicians and policy-makers to make informed recommendations about SSBs and cardiometabolic health, new evidence needs to be considered alongside existing literature. In this review, we provide an overview of global SSB intake trends and an updated summary on the evidence from prospective cohort studies and trials linking SSBs to weight gain and related cardiometabolic conditions including MetSyn. Findings from cross-sectional or case-control studies were not considered since these designs are more prone to confounding and other biases. Biological mechanisms, alternative beverage options, and policy strategies to limit SSB consumption are also discussed.

## 2. SSB Intake Trends

Consumption of SSBs has decreased modestly in the US since around 2002 [13]; however, intake levels are still high and, in some groups, nearly exceed the Dietary Guidelines for Americans’ [14] and World Health Organization’s (WHO) [7] recommendation for no more than 10% of daily calories from all added sugar. National Health and Nutrition Examination Survey (NHANES) data show that US adults consumed an average of 145 kcal/day from SSB, corresponding to 6.5% of total calories, between 2011 and 2014 with higher intake levels reported among younger age groups and among non-Hispanic black and Hispanic men and women [15].

**Figure 1:**
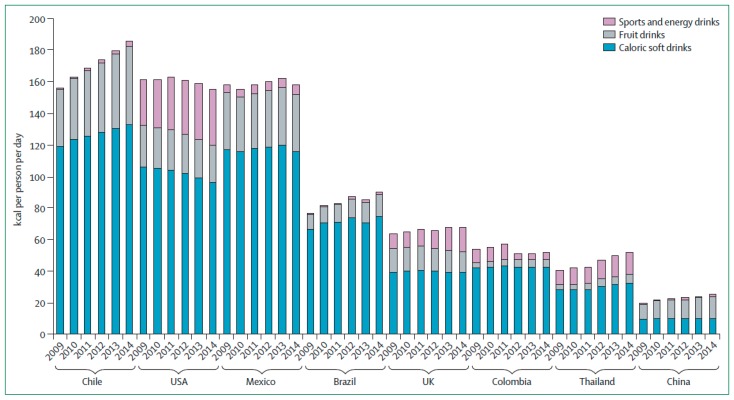
Sales of sugar-sweetened beverages (SSBs) in kcal per person per day by beverage type in 2009–2014 in selected countries. Data from Euromonitor Passport International, which were obtained from nutrition fact panels and websites of sugar-sweetened beverage companies; kcal = kilocalories [18].

In contrast to the US and other high-income countries where consumption of SSBs is either declining or plateauing, intake of SSBs is increasing in many LMICs as a consequence of widespread urbanization and beverage marketing. A report based on survey data from adults in 187 countries found that SSB consumption was higher in upper–middle-income countries and lower–middle-income countries compared to high-income or low-income countries [16]. Of the 21 world regions evaluated, SSB consumption was highest in the Caribbean and lowest in East Asia [16]. Another study among adolescents in 53 LMICs found that soda intake was most frequent in Central and South America, and least frequent in Southeast Asia. Across all populations surveyed, 54% consumed soda at least once a day, and one in five adolescents in Central and South America consumed soda three or more times per day [17]. These trends are supported by another study that reported that per capita sales of SSB (in daily calories per person) increased in most LMICs, while sales declined in some high-income regions, indicative of consumption patterns [18]. Chile was identified as having the highest per capita sales of SSB in 2014, followed by Mexico, the US, Argentina, and Saudi Arabia [18]. The fastest growth in sales of SSB between 2009 and 2014 was seen in Chile, along with China, Thailand, and Brazil [18] (Figure 1). For some regions, disparities in SSB intake tend to track with disparities in obesity and T2D prevalence. For example, in the US, lower socioeconomic status (SES) groups tend to have higher SSB intake levels, and these groups also tend to have a higher risk for developing obesity and T2D.

## 3. Weight Gain and Obesity

Many observational studies have evaluated the relationship between consumption of SSBs and weight gain or obesity. The majority [5,19,20,21,22,23] of systematic reviews and meta-analyses on this topic found positive associations between SSBs and weight gain or risk of overweight or obesity. However, others reported null associations [24]. Our previous meta-analysis, the most comprehensive to date, found that a one-serving-per-day increase in SSB was associated with an additional weight gain of 0.12 kg over one year [5]. In this analysis, we included estimates that were not adjusted for total energy intake since the association between SSBs and weight gain is likely mediated through calories. In addition, all of the studies included in the meta-analysis had repeated measurements of diet and weight, and evaluated weight change in relation to change in SSB intake. This type of analysis strategy has some of the features of a quasi-experimental design, although it lacks the element of randomization. An advantage of this design is the generalizability to a real-world setting, because participants are able to change their diet and lifestyle without investigator-driven intervention. Although the results of the meta-analysis seem modest, adult weight gain in the general population is a gradual process, occurring over decades and averaging about one pound (0.45 kg) per year [25]; thus, small gains in weight from SSBs could be substantial over many years.

The association between SSBs and obesity is strengthened by our previous analysis of gene–SSB interactions [26]. Based on data from three large cohorts, we found that individuals who consumed one or more servings of SSB per day had genetic effects on body mass index (BMI) and obesity risk that were twice as large as those who consumed SSBs less than once per month. These data suggest that regular consumers of SSB may be more susceptible to genetic effects on obesity, or that persons with a greater genetic predisposition to obesity may be more susceptible to the deleterious effects of SSBs on BMI.

Compared to observational studies, most trials have evaluated short-term effects on weight change rather than long-term patterns. In our previous meta-analysis of five trials, we found that adding SSBs to the diet significantly increased body weight [5]. Another meta-analysis of seven randomized controlled trials (RCTs) also found a significant increase in body weight when SSBs were added to the diet [24]. However, in their meta-analysis of eight trials attempting to reduce SSB intake, no overall effect on BMI was observed, but a significant benefit on weight loss/less weight gain was observed among individuals who were overweight at baseline [24]. Of note, this meta-analysis included two of the largest and most rigorously conducted RCTs in children and adolescents [27,28] to date.

Another meta-analysis evaluating the effects of dietary sugars on body weight found that, in trials of adults with ad libitum diets, reducing intake of free sugar or SSB was associated with a decrease in body weight, while increasing intake was associated with a comparable weight increase [23]. Because isoenergetic exchange of dietary sugars with other carbohydrates showed no change in body weight, it seems likely that the change in body weight that occurs with modifying intakes of SSBs is mediated via changes in calories [23]. The majority of studies on SSBs and body weight focused on prevention of weight gain rather than weight loss, which is an important distinguishing factor. From a public health point of view, identifying determinants of weight gain is more impactful than short-term weight loss in reducing obesity prevalence [29]. This is because, once an individual develops obesity, it is difficult to achieve and maintain weight loss. For this reason, fewer studies have evaluated the impact of SSB restriction on weight loss.

## 4. Metabolic Syndrome and Risk Factors

Few prospective studies have examined intake of SSBs in relation to the development of MetSyn, most likely due to challenges in outcome assessment. However, these along with studies of individual risk factors generally show adverse associations that are consistent with studies linking SSBs to weight gain and risk of T2D. Our previous meta-analysis of three cohort studies found a higher risk of about 20% (relative risk (RR), 1.20; 95% confidence interval (CI), 1.02–1.42) comparing highest to lowest categories of SSB intake [6]. However, a recent meta-analysis of three cohort studies by Narain et al. found a marginal positive association between intake of SSB and risk of MetSyn [30]. The discrepancy may be due to inclusion of different studies. The more recent meta-analysis included a new study among children and adolescents [31], which was combined with studies in adults and excluded a study from the Multi-Ethnic Study of Atherosclerosis (MESA) cohort [32], which we included. We also included the cohort-wide estimate from the Framingham Heart study that combined diet and regular soft drinks, while Narain et al. used an estimate from a sub-group with regular soft drink consumption but limited power [33]. Recent studies not included in these meta-analyses have also found positive associations. A study in the Prevención con Dieta Mediterránea (PREDIMED) trial found a positive association between SSBs and fruit juice with MetSyn among participants at high risk for CVD, but cautioned that associations should be interpreted conservatively due to low intake levels [34]. In a cohort of healthy Korean adults, a positive association between SSB and MetSyn was observed in women but not men [35]. According to the authors, the sex difference could be due to the action of sex hormones. Some studies of MetSyn found marginal associations with SSBs; however, because they adjusted for total energy intake, the results may have been underestimated [32,36].

Studies examining individual risk factors rather than MetSyn tend to be more consistent. In the Coronary Artery Risk Development in Young Adults (CARDIA) study, higher SSB consumption was associated with a number of cardiometabolic outcomes: high waist circumference (RR: 1.09; 95% CI 1.04, 1.14), high low-density lipoprotein (LDL) cholesterol (RR: 1.18; 95% CI 1.02, 1.35), high triglycerides (RR: 1.06; 95% CI 1.01, 1.13), and hypertension (RR: 1.06; 95% CI 1.01, 1.12) [37]. Although central adiposity is a risk factor for CVD independent of body weight, few cohort studies have examined this relationship with SSBs, likely due to challenges in measurement. In the Mexican Teacher’s cohort, compared to no change, increasing soda consumption by one serving per day was associated with a ~1-cm increase in waist circumference (0.9 cm; 95% CI = 0.5, 1.4) over two years [38]. Similar findings were observed in a Spanish cohort [39]. Both of these studies used waist circumference as a proxy for central adiposity. However, waist circumference does not distinguish between different types of abdominal fat accumulation, e.g., visceral vs. subcutaneous adipose tissues, which may be differently associated with cardiometabolic risk. In the Framingham Third Generation cohort, Ma and colleagues found that SSB intake was associated with a long-term adverse change in visceral adiposity as measured by abdominal computed tomography scan (i.e., increased visceral adipose tissue (VAT) volume and decrease in VAT attenuation), independent of weight gain [40].

In a systematic review including five prospective cohort studies examining SSB intake in relation to vascular risk factors, positive associations were observed for blood pressure, triglycerides, LDL cholesterol, and blood glucose, and an inverse association was observed for high-density lipoprotein (HDL) cholesterol [12]. These findings were supported by cross-sectional analyses in the Health Professionals Follow-up Study (HPFS) and Nurses’ Health Study (NHS) cohorts that found associations between SSB and higher plasma triglycerides, along with inflammatory cytokines and other cardiometabolic risk factors [9,41]. Accumulating evidence also suggests a role of SSBs in the development of hypertension [42,43,44]. A meta-analysis of six cohort studies found that a one serving/day increase in SSB intake was associated with ~8% higher risk of hypertension (RR: 1.08, 95% CI: 1.06, 1.11) [42]. Similar results were reported in two previous meta-analyses [43,44]. Regular consumption of SSBs was also associated with hyperuricemia and with gout [45,46].

Findings from short-term trials and experimental studies also provide important evidence linking SSBs with cardiometabolic risk factors, and they provide mechanistic support for the epidemiologic evidence linking intake of SSBs to higher risk of T2D and coronary heart disease (CHD). Many of these studies explored the effects of sugars used to flavor SSBs such as high-fructose corn syrup (HFCS) (~42–55% fructose, glucose and water) or sucrose (50% fructose and glucose) in liquid form. A meta-analysis of 39 RCTs found that higher compared to lower intakes of dietary sugars or SSB significantly raised triglyceride concentrations (mean difference (MD): 0.11 mmol/L; 95% CI: 0.07, 0.15), total cholesterol (MD: 0.16 mmol/L; 95% CI: 0.10, 0.24), LDL cholesterol (MD: 0.12 mmol/L; 95% CI: 0.05, 0.19), and HDL cholesterol (MD: 0.02 mmol/L; 95% CI: 0.00, 0.03) [47]. The most pronounced effects were noted in studies that ensured energy balance and when no difference in weight change was reported, suggesting that the effects of SSBs on lipids are independent of body weight [47]. This meta-analysis also found a significant blood-pressure-raising effect of sugars, particularly in studies ≥8 weeks in duration (MD 6.9 mm Hg (95% CI: 3.4, 10.3) for systolic blood pressure, and 5.6 mm Hg (95% CI: 2.5, 8.8) for diastolic blood pressure) [47].

In a two-week parallel-arm trial, Stanhope and colleagues showed that consuming beverages containing 10%, 17.5%, or 25% of energy requirements from HFCS produced a significant linear dose–response increase in postprandial triglycerides, fasting LDL cholesterol, and 24-h mean uric acid concentrations [48]. In another study, uric acid was found to increase after six months of consuming 1 L/day of sucrose-sweetened cola compared to isocaloric consumption of milk, water, or diet beverages [49]. The change in uric acid correlated with changes in liver fat (*p* = 0.005), triglycerides (*p* = 0.02), and insulin (*p* = 0.002) [49] In a 10-week trial among overweight healthy participants, consuming a sucrose-rich diet compared to a diet rich in artificial sweeteners, significant increases in postprandial glycemia, insulinemia, and lipidemia were observed [50]. A randomized crossover trial among normal-weight healthy men found that, after three weeks, SSBs consumed in small to moderate quantities resulted in impaired glucose and lipid metabolism and promoted inflammation [51]. Other trials have found inconsistent results on markers of inflammation, which may be due to differences in study duration. [52,53].

## 5. Diabetes and CVD

**Figure 2:**
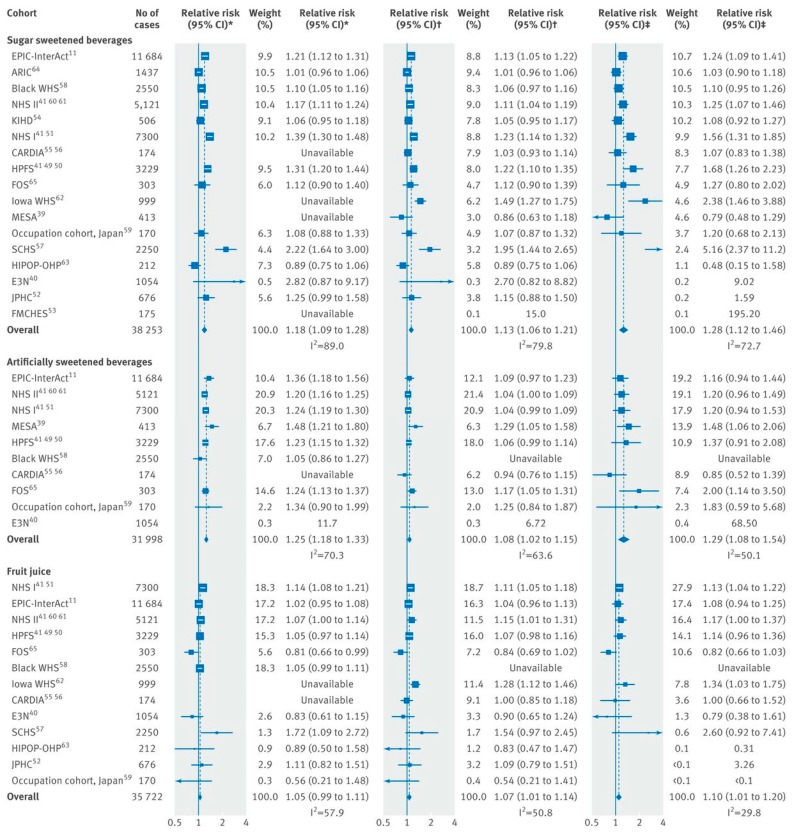
Prospective associations for an incremental increase in beverage consumption with incident type 2 diabetes (T2D): random effects meta-analysis. * Unadjusted for adiposity; † adjusted for adiposity; ‡ adjusted for adiposity and within person variation [54].

Although experimental evidence from RCTs is lacking due to high cost and other feasibility considerations, findings from prospective cohort studies have shown strong and consistent associations in well-powered studies. A meta-analysis of 17 prospective cohort studies evaluating SSB consumption and risk of T2D found that a one-serving-per-day increment in SSB was associated with an 18% higher risk of T2D (95% CI: 9% to 28%) among studies that did not adjust for adiposity [54] (Figure 2). Among studies that adjusted for adiposity, the estimate was attenuated to 13% (6% to 21%), suggesting a partial mediating role of adiposity in this association. Positive yet weaker associations were also noted for juice and artificially sweetened beverages (ASB). This study also estimated the population attributable fraction for T2D from consumption of SSB in the US and United Kingdom (UK). Based on their estimates, 8.7% (95% CI, 3.9% to 12.9%) of T2D cases in the US and 3.6% (95% CI, 1.7% to 5.6%) in the UK would be attributable to the consumption of SSBs [54]. These results, which are consistent with previous meta-analyses [6,55,56], confirm that the consumption of SSBs is associated with increased risk of T2D independently of adiposity and suggests that the consumption of SSBs over many years could be related to a substantial number of new cases. Recent studies in the Mexican Teacher’s cohort [57] and Northern Manhattan study [58], a multi ethnic urban cohort in New York City, provide additional support linking intake of SSBs to risk of T2D, and have expanded the generalizability of the findings across different populations.

Emerging evidence linking intake of SSBs to CVD is strengthened by consistent associations of SSBs with cardiometabolic risk factors, in addition to weight gain and risk of T2D. A meta-analysis of nine prospective cohort studies found that a one-serving-per-day increase in SSB was associated with a 13% higher risk of stroke (RR: 1.13, 95% CI: 1.02, 1.24) based on one study, and 22% higher risk of myocardial infarction (MI) (RR: 1.22, 95% CI: 1.14, 1.30) based on two studies [10]. In the categorical analysis comparing high vs. low SSB intake, there was a 19% higher risk of MI (RR: 1.19, 95% CI: 1.09, 1.31) based on three studies, but no significant association was observed for stroke (three studies) [10]. For the association with stroke, moderate heterogeneity was evident. After stratification by sex and stroke type, the pooled results suggested that women who consume SSBs have a higher risk of ischemic stroke (RR: 1.33, 95% CI: 1.07, 1.66), while no differences were noted for men or for men and women with hemorrhagic stroke [10]. These findings are consistent with a previous meta-analysis of four prospective cohort studies, which found a 17% higher risk of CHD (95% CI: 7% to 28%) comparing extreme SSB intake categories and a 16% higher risk of CHD per one-serving-per-day increment (10% to 23%) [11]. Similar to studies of T2D, when estimates that did not adjust for BMI or energy intake were included in the meta-analysis, the magnitude of the association increased (RR: 1.26, 95% CI: 1.16, 1.37), suggesting these factors as partial mediators of the association. A systematic review by Keller et al. also reported positive associations between SSB and CHD but noted that associations were only apparent in large studies with long durations of follow-up [12]. This review also found that, among studies that evaluated SSB intake in relation to risk of stroke, positive associations were observed only among women.

Building on the clinical evidence, a few studies have also shown a link between SSB intake and risk of all-cause or CVD mortality. We recently found that, among over 118,000 women and men from the NHS and HPFS, intake of SSBs was positively associated with risk of death from any cause in a dose-dependent manner [59]. Compared with drinking SSBs less than once per month, drinking one to four per month was linked with a 1% higher risk, two to six per week with a 6% higher risk, one to two per day with a 14% higher risk, and two or more per day with a 21% higher risk [59]. The higher risk of death associated with SSBs was more pronounced among women than men and was driven by CVD mortality. Compared with infrequent SSB consumers, those who consumed two or more per day had a 31% higher risk of death from CVD [59]. These findings are consistent with a previous study conducted in a prospective analysis of NHANES, which found a 29% higher risk of CVD mortality (RR: 1.29, 95% CI: 1.04, 1.60) comparing participants who consumed seven or more servings of SSBs per week to those who consumed one serving per week or less [60]. It was also estimated in NHANES that 7.4% of all cardiometabolic deaths in the US could be attributed to intake of SSBs in 2012 [61]. More recently, in the US-based Reasons for Geographic and Racial Differences in Stroke (REGARDS) study, each additional 12-oz serving/day of SSBs was associated with an 11% higher risk of all-cause mortality [62]. However, no association was observed for risk of death from CHD, which may have been due to a limited number of cases. In contrast, results from a cohort of Chinese adults in Singapore [63] and an elderly population in the US [64], both with very low intake levels, found no significant association between SSBs and mortality.

## 6. Biological Mechanisms

SSBs contribute to weight gain through decreased satiety and an incomplete compensatory reduction in energy intake at subsequent meals following ingestion of liquid calories [20]. A typical 12-oz (360 mL) serving of soda contains ~140–150 calories and ~35–37.5 g of sugar. If these calories are added to the diet without compensating for the additional calories, one can of soda per day could in theory lead to a weight gain of five pounds in one year [65]. Short-term feeding trials that show greater energy intake [66] and weight gain [50,66,67,68,69] from consuming SSBs compared to ASBs indirectly illustrate this point. While few studies have evaluated this mechanism, some evidence supporting incomplete compensation for liquid calories has been provided by studies showing greater energy intake and weight gain after isocaloric consumption of beverages compared to solid food [70,71,72]. These studies suggest that calories from sugar in liquid beverages may not suppress intake of solid foods to the level needed to maintain energy balance; however, the mechanisms responsible for this response are largely unknown.

**Figure 3:**
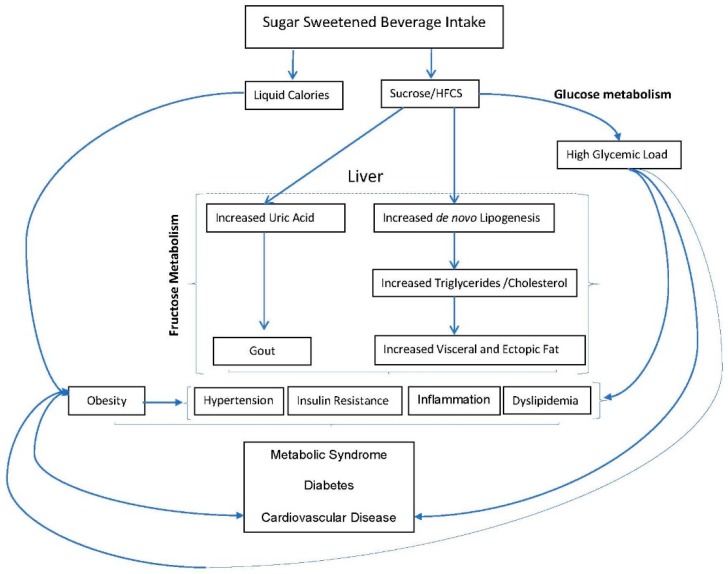
Biological mechanisms linking intake of sugar-sweetened beverages (SSB) to the development of obesity, metabolic syndrome (Met Syn), diabetes, and cardiovascular disease (CVD). Incomplete compensation for liquid calories leads to obesity, which is a risk factor for cardiometabolic outcomes. Increased diabetes, MetSyn, and CVD risk also occur independent of weight through development of risk factors precipitated by adverse glycemic effects and increased fructose metabolism in the liver. Excess fructose ingestion promotes hepatic uric acid production, de novo lipogenesis, and accumulation of visceral and ectopic fat, and also leads to gout. HFCS = high-fructose corn syrup.

SSBs contribute to the development of T2D and cardiometabolic risk in part through their ability to induce weight gain, but also independently through metabolic effects of constituent sugars (Figure 3). Consumption of SSBs has been shown to induce rapid spikes in blood glucose and insulin levels [73,74]. As such, these beverages have moderate-to-high glycemic index (GI) values [75], which, in combination with the large quantities consumed, contribute to a high dietary glycemic load (GL). High-GL diets can promote insulin resistance [76], exacerbate inflammatory biomarkers [77], and are associated with higher risk of T2D [78,79] and CHD [80]. Consuming fructose from SSBs as a component of sucrose or HFCS may further impact cardiometabolic risk. Fructose alone is poorly absorbed but is enhanced by glucose in the gut, thus accounting for the rapid and complete absorption of both fructose and glucose when ingested as sucrose or HFCS. Fructose, when consumed in moderate amounts, is metabolized in the liver where it is converted to glucose, lactate, and fatty acids to serve as metabolic substrates for other cells in the body [81]. When consumed in excess, this can lead to increased hepatic de novo lipogenesis, atherogenic dyslipidemia, and insulin resistance. The increase in hepatic lipid promotes production and secretion of very-low-density lipoproteins (VLDLs) leading to increased concentrations of postprandial triglycerides. Consumption of fructose-containing sugars is associated with production of small dense LDL cholesterol, which may be due to increased levels of VLDL-induced lipoprotein remodeling [48,82]. Fructose was also shown to promote the accumulation of VAT and the deposition of ectopic fat [83,84,85,86], processes indicative of cardiometabolic risk. Accumulating evidence suggests that the metabolic effects of fructose may be modified by physical activity level with more adverse effects observed under conditions of high fructose intake and low levels of physical activity [87]. According to this model, the adverse metabolic effects of fructose would occur when fructose intake chronically exceeds the capacity of the liver to release lactate and glucose for muscle, i.e., when there is a mismatch between fructose intake and energy output in the muscle. Fructose is the only sugar known to increase production of uric acid [87]. The production of uric acid in the liver has been shown to reduce endothelial nitric oxide, which may be implicated in the association between SSBs and CHD [88]. Hyperuricemia often precedes development of obesity and T2D, and clinical evidence suggests that hyperuricemia may mediate the association between SSB consumption and hypertension through the development of renal disease, endothelial dysfunction, and activation of the renin–angiotensin system [88]. In addition, hyperuricemia is associated with the development of gout [45,46], and gout and hyperuricemia are associated with hypertension, T2D, MetSyn, kidney disease, and CVD [88,89].

## 7. Alternative Beverages

Several beverages have been suggested as alternatives to SSBs including water, 100% fruit juice, coffee, tea, and ASBs. Unlike SSBs, water does not contain liquid calories and, for most people with access to safe drinking water, it is the optimal calorie-free beverage. We found that replacement of one serving per day of SSBs with one serving of water was associated with less weight gain [90] and a lower risk of T2D [91]. With more consumers opting for water, several types of sparkling and flavored waters have emerged on the market, which may make switching to water more feasible for habitual SSB consumers.

Although 100% fruit juice might be perceived as a healthy choice since juice contains some vitamins and nutrients, they also contain a relatively high number of calories from natural sugars. Previous cohort studies have found positive associations between consumption of fruit juice and weight gain [92] and T2D [93], while the opposite has been shown for whole fruit [25,94]. Sugars in juice are absorbed more quickly than those in fruit and vegetables, which are absorbed more slowly due in part to their fiber content [95,96]. The rapid absorption of liquid fructose (from juice) compared to solid forms is more likely to result in higher concentrations of fructose in the liver and increase the rate of hepatic extraction of fructose, de novo lipogenesis, and production of lipids [97,98]. A recent study in the REGARDS cohort found that fruit juice intake was associated with a higher risk of all-cause mortality [62]. However, other studies have shown benefits of juice on cardiometabolic markers [92,99]. This suggests a need for further research that can evaluate different types of juice, since the nutrient profile and sugar content across various juices may differ. Nonetheless, based on the current evidence, it is recommended that daily intake of fruit juice be limited to 8 oz for adults.

Numerous studies have shown that regular consumption of coffee (decaffeinated or regular) and tea can have favorable effects on T2D and CVD risk [100,101], possibly due to their high polyphenol content. These beverages can thus be considered healthful alternatives to SSBs for individuals without contraindications, provided that caloric sweeteners and creamers are used sparingly, and that intake does not exceed the guidelines for caffeine. We found that substituting one serving per day of SSBs with one cup of coffee was associated with a 17% lower risk of T2D [102].

ASBs provide few to no calories but retain a sweet flavor, making them an attractive alternative to SSBs. Paradoxically, some cohort studies have reported positive associations between ASB consumption and weight gain and risk of T2D and CVD [32,36]. These findings may be due in part to residual confounding by unmeasured or poorly measured lifestyle factors or reverse causation, since individuals with obesity or metabolic risk may switch to ASBs for health reasons, which can result in spurious associations between ASBs and cardiometabolic outcomes. Studies with repeated measurements of diet, which are less prone to reverse causation, have shown only marginal nonsignificant associations with ASBs [8,9,25,59,102]. Cohort-based substitution analysis has also shown inverse associations with weight gain, T2D and mortality with replacement of SSBs with ASBs [59,90,91]. In addition, short-term trials that assessed ASBs as a replacement for SSBs reported modest benefits on body weight and metabolic risk factors [5,103]. On the other hand, some mechanisms have been proposed linking ASBs to adverse cardiometabolic health such as the intense sweetness of artificial sweeteners conditioning toward a preference for sweets or stimulating a cephalic insulin response, and more recently through alterations in gut microflora linked to insulin resistance [104]. However, these mechanisms are not well understood, and different types of artificial sweeteners may have different metabolic effects.

Consumption of ASBs in place of SSBs could be a helpful strategy to reduce cardiometabolic risk among heavy SSB consumers with the ultimate goal of switching to water or other healthful beverages. Further studies are needed to evaluate potential metabolic consequences of consuming ASBs over the life course and better understand underlying biological mechanisms. Understanding potential health impacts of ASB consumption is especially important in the context of sugar reduction policies such as taxation and labeling, which may lead to product reformulation and more ASBs in the food supply.

## 8. Policy Considerations

In response to the strong evidence linking consumption of SSBs to weight gain and risk of T2D and CVD, national and international organizations are already calling for reductions in intake of these beverages to help curb obesity and improve cardiometabolic health [105]. Both the WHO and 2015–2020 US Dietary Guidelines recommend an upper limit of 10% of total energy from added sugar, and numerous associations specifically recommend limiting intake of SSBs. In addition to widespread public health recommendations, public policies are needed to change consumption pattern at the population level (Box 1). The most common actions implemented to reduce SSB consumption include taxation, reduction of availability in schools, restrictions on marketing to children, public awareness campaigns, and front-of-package labelling [18,106]. Several cities in the US and globally have implemented excise taxes on SSBs as a strategy to reduce intake levels and generate revenue to support various public efforts. The most rigorously evaluated SSB tax to date is in Mexico, where a nationwide excise tax of 10% (one peso per liter) was implemented in 2014. Two years after the tax was implemented, a net decrease of 7.6% in sales of sugary drinks was observed, while sales of untaxed beverages such as water increased by 2.1% [107]. It was estimated that, between 2013 to 2022, the tax alone will prevent nearly 200,000 cases of obesity and save $980 million in direct healthcare costs, with the majority of benefits in young adults [108]. In Berkeley, California, the first US city to levy a penny-per-ounce excise tax on SSBs, sales of SSBs fell 9.6%, while sales of untaxed beverages, such as water and milk, increased 3.5%, comparing pre-tax to one-year-post-implementation trends [109]. Whether these early benefits of the tax will continue over the long term and translate into improvements in health will be important factors to monitor over time. In the US, the recently revised nutrition facts label will now require manufacturers (compliance by 1 January2020 to 1 July 2021, depending on annual food sales) to disclose the added sugar content of products, and will be accompanied by a percent daily value, with a goal of helping consumers make healthier choices. To achieve meaningful changes in beverage consumption patterns, a combination of multiple strategies will be needed, together with consumer education, and will serve as important steps in changing social norms surrounding beverage habits. Implementing and evaluating these policy actions in relation to behavior changes in the short term and clinical outcomes in the long term should remain a priority for scientists and policy-makers.

Box 1 **Policy strategies to reduce consumption of sugar-sweetened beverages (SSBs).**

- Governments should impose financial incentives such as taxation of SSBs of at 1east a 10% price increase, and implement limits for use of Supplemental Nutrition Assistance Program (SNAP) benefits for SSBs or subsidizing SNAP purchases of healthier foods, to encourage healthier beverages choices.
- Regulations are needed to reduce exposure to marketing of unhealthy foods and beverages in the media and at sports events or other activities, particularly in relation to children.
- Front-of-package labelling or other nutrition labeling strategies should be implemented to help guide consumers to make healthy food and beverage choices. These changes should be accompanied by concurrent public health awareness campaigns.
- Policies should be adopted to reduce the availability of SSBs in the workplace, healthcare facilities, government institutions, and other public spaces, and ensure access to safe water and healthy alternatives. Policies that make healthful beverages the default choice should also be adopted.
- Educational campaigns about the health risks associated with overconsumption of SSBs should be aimed at healthcare professionals and clinical populations.
- National and international campaigns targeting obesity and chronic disease prevention should include the health risks associated with overconsumption of SSBs.
- National and international dietary recommendations should include specific guidelines for healthy beverage consumption.

## 9. Conclusions

Intake of SSBs remains high in the US and is rising in many parts of the world. Based on findings from prospective cohort studies and short-term experimental trials of cardiometabolic risk factors, there is strong evidence for an etiological relationship between intake of SSBs and weight gain and risk of T2D and CHD. The evidence for a link with stroke is less clear and warrants further research, including the potential sex difference. Few studies have investigated intake of SSBs in relation to MetSyn, and this may be due to challenges in assessment and controversy about its clinical utility. However, findings on individual risk factors suggest a link. Since development of MetSyn often precedes onset of T2D and CHD, preventing or reversing MetSyn could be an effective way to curtail rising T2D and CHD rates.

SSBs are thought to promote weight gain through incomplete compensation for liquid calories at subsequent meals. These beverages may increase T2D and CHD in part through weight gain and independently through metabolic effects of constituent sugars. A mechanistic area that warrants future research is exploring the health effects of sugar consumed in solid form compared to SSB, and further elucidating compensatory effects of liquid vs. solid sugars. With the strength of evidence sufficient to call for reductions in intake of SSB for optimal cardiometabolic health, important research gaps exist regarding suitable alternative beverages, including the long-term health effects of consuming ASBs. Continued evaluation of SSB policies that are already in place is needed, as are more and higher-quality RCTs to identify effective strategies to reduce intake of SSBs at the individual and population level. SSBs present a clear target for health policy; however, chronic disease prevention should focus on improving overall diet quality by consuming more healthful foods and limiting unhealthy ones. Given the high levels of intake across the globe, reducing consumption of SSBs is an important step in improving diet quality that could have a measurable impact on weight control and improving cardiometabolic health.
